# Investigation of modified platelet-rich plasma (mPRP) in promoting the proliferation and differentiation of dental pulp stem cells from deciduous teeth

**DOI:** 10.1590/1414-431X20165373

**Published:** 2016-09-01

**Authors:** J. Wen, H.T. Li, S.H. Li, X. Li, J.M. Duan

**Affiliations:** 1 Guangdong Provincial Stomatological Hospital, Guangzhou, Guangdong Province, China; 2 Department of Stomatology, Guangzhou General Hospital, Guangzhou Military Command, Guangzhou, Guangdong Province, China; 3 Department of Stomatology, Zhongshan City People's Hospital, Zhongshan, Guangdon Province, China

**Keywords:** mPRP, SHEDs, ALP, RUNX2, OCN

## Abstract

Stem cells from human exfoliated deciduous teeth (SHEDs) have great potential to treat various dental-related diseases in regenerative medicine. They are usually maintained with 10% fetal bovine serum (FBS) *in vitro*. Modified platelet-rich plasma (mPRP) would be a safe alternative to 10% FBS during SHEDs culture. Therefore, our study aimed to compare the proliferation and differentiation of SHEDs cultured in mPRP and FBS medium to explore an optimal concentration of mPRP for SHEDs maintenance. Platelets were harvested by automatic blood cell analyzer and activated by repeated liquid nitrogen freezing and thawing. The platelet-related cytokines were examined and analyzed by ELISA. SHEDs were extracted and cultured with different concentrations of mPRP or 10% FBS medium. Alkaline phosphatase (ALP) activity was measured. Mineralization factors, RUNX2 and OCN, were measured by real-time PCR. SHEDs were characterized with mesenchymal stem cells (MSCs) markers including vimentin, CD44, and CD105. mPRP at different concentrations (2, 5, 10, and 20%) enhanced the growth of SHEDs. Moreover, mPRP significantly stimulated ALP activity and promoted expression of RUNX2 and OCN compared with 10% FBS. mPRP could efficiently facilitate proliferation and differentiation of SHEDs, and 2% mPRP would be an optimal substitute for 10% FBS during SHEDs expansion and differentiation in clinical scale manufacturing.

## Introduction

Stem cells have shown high potential in dental-related regenerative medicine. Three critical factors, including seeding cells, scaffold materials, and osteogenesis-related differentiation factors, determine the effect and efficiency of tissue regeneration. Mesenchymal stem cells (MSCs) are considered ideal seeding cells for bone regeneration (1). A series of studies have demonstrated that bone marrow MSCs (BMMSCs) can differentiate into multiple cell types, such as skeletal tissue, adipocytes, and osteoblasts (2). However, BMMSCs extraction is still a complex process for oral tissue engineering. Thus, dental pulp stem cells (DPSCs) became a safer and easier substitute for BMMSCs. DPSCs were first isolated by Gronthos et al., and became a good candidate for tissue engineering (3). Other similar DPSCs include stem cells from human exfoliated deciduous teeth (SHEDs) first isolated by Miura et al., in 2003 (4). Compared with BMMSCs and DPSCs, SHEDs have the highest proliferative capacity and multilineage differentiation potential (4,5).

To culture the extracted SHEDs *in vitro*, fetal bovine serum (FBS) is the most commonly used culture supplement for MSCs, with multiple nutrition and growth factors, such as platelet-derived growth factors (6), insulin-like growth factors I and II (7), and TGF-β (8). Nevertheless, compositions of FBS are too complex and may cause unexpected problems such as immunological rejection, infections by bovine virus and other pathogens, which limit its application in clinical trials (9). Platelet rich plasma (PRP), which is blood plasma enriched with platelets from autologous whole blood without exogenous antigens, could be a safe culture supplement and solve the above technical disadvantages (10). PRP contains a cocktail of growth factors, such as platelet-derived growth factor (PDGF), basic fibroblast growth factor (bFGF), insulin-like growth factor, transforming growth factor β (TGF-β), and vascular endothelial growth factor. All these growth factors play an important role in supporting and stimulating MSCs growth and expansion (9,11,12). PRP has been widely applied in the field of oral regenerative treatment, such as maxillofacial bone defect repair and guided periodontal tissue regeneration (10). During treatment, SHEDs mixed with PRP are implanted into mandible defect areas to promote osseointegration and vascularization. A series of studies have confirmed that PRP can promote proliferation, migration and differentiation of mesenchymal stem cells, reduce the time of cell fusion, increase sizes of cell colony-forming units, maintain stem cells osteogenic, chondrogenic and adipogenic differentiation capacity, and maintain an immunosuppressive state (10,13,14). Flow cytometry assays also showed that MSCs expressed high levels of PDGF-A, PDGF-B, bFGF, TGF-β and IGF-1 receptors, suggesting their functional importance in MSCs maintenance (15,16).

When DPSCs are cultured with PRP, its immunophenotype, colony formation unit (CFU) and directional differentiation ability remain unchanged. PRP promotes the proliferation and protein synthesis of DPSCs through PI3K/AKT, MAPK and NFκB signaling pathways activation (17). Furthermore, PRP induces DPSCs mineralization through upregulation of osteogenic genes and osteopontin (OPG) protein, enhancing alkaline phosphatase (ALP) activity (18).

Therefore, our hypothesis is that modified (m)PRP could be a safe alternative to FBS to promote SHEDs proliferation through multiple platelet-derived growth factors. In the present study, we successfully separated the mPRP and SHEDs with high purity. Thereafter, we evaluated the effects of different concentrations of mPRP on the proliferation and differentiation of SHEDs in comparison with 10% FBS. Furthermore, we studied multiple differentiation phenotypes and factors when SHEDs were supplied with differentiation medium containing different concentrations of mPRP and 10% FBS.

## Material and Methods

All experimental procedures were carried out according to hospital regulations and medical ethics standards. Platelets: human platelets were collected from 4 male volunteers in the Blood Transfusion Department, Guangzhou General Hospital, Guangzhou Military Command. The volunteers were AB blood type, 18–35 years old, had good health status, and no family disease history. All volunteers gave written informed consent.

### Separation and activation of PRP

Isolation of improved PRP: 10 mL platelets were extracted from volunteers and centrifuged at 1000 *g* for 20 min at room temperature to discard the supernatant. Heparin was added to platelets to a final concentration of 2 U/mL. Four samples were mixed thoroughly and counted by a Hematology Analyzer (BC-3000, Shenzhen Mindray Bio-Medical Electronics Co., LTD, China). The platelet concentration was adjusted to approximately 10^12^/L for PBS and for mPRP. Improved PRP activation: PRP was aliquoted into vials, immersed in liquid nitrogen for 5 min and quickly warmed at 37°C for 5 min, three times (10). Vials were then centrifuged at 1000 *g* for 20 min at room temperature and platelet sediment was collected through a 0.2 μm filter. The samples were stored at –80°C until use.

### Measurement of PDGF-AA and TGF-β1 by ELISA

The levels of PDGF-AA and TGF-β1 were measured by ELISA kit (R&D, USA). Serial dilution of PDGF-AA and TGF-β1 were prepared on a 96-well plate, for standardization. The concentrations of the growth factors were determined based on a standard curve. Each test was done with triplicate wells.

### Isolation and culture of SHEDs

*Teeth*. After parents signed the informed consent form, two mandibular caries-free lower central deciduous incisors from 6- to 10-year-old healthy children were extracted by a dentist. The pulp tissue was removed and cultured to obtain dental pulp stem cells according to the literature (4). Briefly, the teeth were placed in precooled α-MEM (Gibco, USA) immediately after removal and sterilized in 75% ethanol. To extract the primary stem cells, high-speed dental handpieces were used along the cementoenamel junction to grind a groove without breaking through to the pulp, within 4 h. After 75% ethanol disinfection and repeated PBS washing, the deciduous incisors were split along the groove. Pulp tissue was extracted with a barbed broach, cut into pieces and digested with 1:1 3 g/L collagenase and 4 g/L neutral protease at 37°C for 1 h. Dental pulp stem cells pellets were suspended with α-MEM, 20% FBS. Cells (2×10^5^) were seeded onto a 6-well plate and cultured in 37°C in a 5% CO_2_ incubator (Heraeus, Germany) for 3 days. α-MEM with 20% FBS was applied to the culture of separated SHEDs for two passages then changed to 10% FBS. When the cells reached 90% confluence, they were transferred to a T25 flask. SHEDs were passed for 3 to 4 times and each passage was determined when reaching 70% confluence.

### Cell proliferation assay

Third passage SHEDs (2×10^3^) were seeded on 96-well plates and cultured for 1 to 7 days. A cell growth curve was drawn based on a CCK-8 cell counting kit (Dojindo, Japan) by measuring 450 nm absorbance. To determine the effect of different concentrations of PRP on SHEDs proliferation, the fourth passage of SHEDs was seeded on 96-well plate supplemented with α-MEM containing 2, 5, 10, and 20% PRP or 10% FBS. The cells were then cultured for 7 days. Everyday, 10 μL CCK-8 was added and absorbance at 450 nm was measured.

### Characterization of SHEDs

*SHEDs morphology observation*. Third passage SHEDs were digested and diluted to the concentration of 1×10^7^/L. Cells were seeded on 6-well plates with polylysine pre-treated coverslips until they adhered onto the slips. Slips were removed and the cells were stained with hematoxylin and eosin to observe cell morphology under a microscope.

*Immunocytochemical detection and phenotypic characterization*. Third passage SHEDs on coverslips were fixed with 4% formalin for 2 h. To eliminate endogenous peroxidase activity, the coverslip was incubated with 10% hydrogen peroxide for 10 min. After blocking with 3% normal goat serum for 30 min, the fixed cells were stained with mouse anti-human cytokeratin (or vimentin) antibody, followed by treatment with the biotinylated secondary antibody. Then, the cells were stained with hematoxylin, dehydrated with xylene, and sealed with neutral gum. The cell phenotype was detected with DAB staining and observed under a microscope.

*Flow cytometry assay*. Fourth passage SHEDs were fixed and stained with CD34, CD44, and CD105 antibodies to check SHEDs surface markers.

### SHEDs *in vitro* differentiation

Third passage SHEDs (1×10^5^) were seeded onto 6-well plates. When cells reached a 70% confluence, cell mineralized nodules and lipid droplets formation capacity were tested after adding mineralization-inducing medium (final concentration of 50 μg/L ascorbic acid, 10 mmol/L β-glycerophosphate and 0.01 mmol/L dexamethasone) or adipogenic induction medium (final concentration of 1 μmol/L dexamethasone, 10 μmol/L insulin, 200 μmol/L indomethacin, 0.5 mmol/L-isobutyl-methylxanthine). After culturing for 30 days, cells were fixed with formalin and stained with Alizarin red or oil red-O to separately test the mineralization and adipogenesis capacities of SHEDs.

### ALP enzyme activity test

Fourth passage SHEDs were seeded (5×10^3^) onto 96-well plates. Every 2, 4, and 6 days, ALP kit (Jiancheng, China) was applied and 520 nm absorbance was measured for the detection of ALP activity using the Enzyme Activity Reader (Biocell, USA).

### RNA extraction and real-time PCR

Total RNA was extracted by Trizol. cDNA was prepared through reverse transcription. Primers were synthesized by Shanghai Shenggong, China. PCR primer sequences are as follows: hRUNX2, forward: 5′- TCCACACCATTAGGGACCATC-3′, reverse: 5′-TGCTAATGCTTCGTGTTTCCA-3′; OCN, forward: 5′-GGCAGCGAGGTAGTGAAGAGA-3′, reverse: 5′-CTCCTGAAAGCCGATGTGG-3′; hGAPDH, forward: 5′-GACAACTTTGGCATCGTGGA-3′, reverse: 5′-ATGCAGGGATGATGTTCTGG-3′. GAPDH was set as the internal control. After the real-time PCR, RUNX2 and OCN mRNA levels were calculated and compared among the different SHEDs groups.

### Data analysis

Experiments were repeated three times and statistical analysis was carried out using GraphPad (GraphPad Software Inc., USA) or SPSS software (SPSS Inc., USA). One-way ANOVA was used to test the difference among different groups in each time-point. To test the interaction between time and groups, two-way factorial ANOVA was applied. Multiple comparisons were based on the LSD method. The results were considered to be significant when P≤0.05.

## Results

### Phenotypic characterization showed that SHEDs are similar to MSC

**Figure 1:**
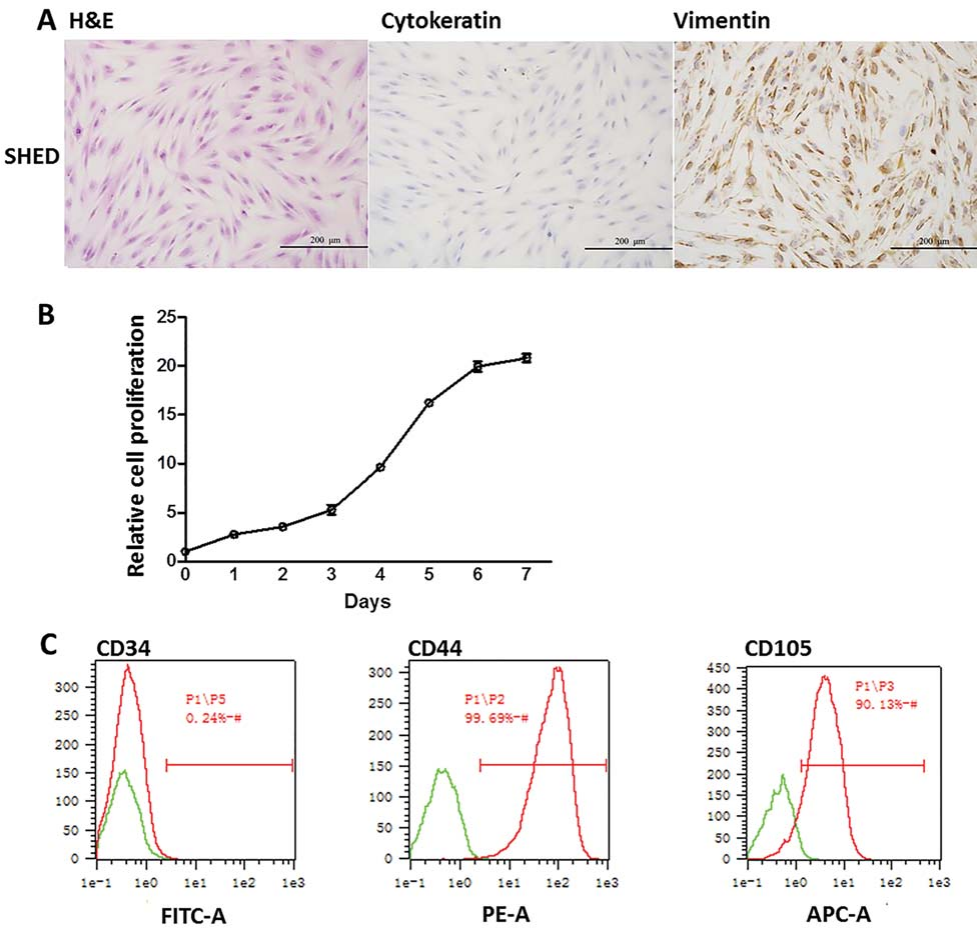
Photomicrographs of extracted stem cells from human exfoliated deciduous teeth (SHEDs) showing characteristics of mesenchymal stromal cells (spindle-like shape) with high proliferation activity. *A*, H&E staining of SHEDs and immunocytochemical staining of cell markers cytokeratin, vimentin, ×200. *B*, SHEDs cell proliferation was quantified by CCK-8 assay for 1-7 days. Data are reported as mean percentages ± SD from three independent experiments. *C*, Flow cytometry analysis of cell surface markers CD34, CD44, and CD105.

HE staining of SHEDs showed that the major morphology of SHEDs was typically spindle-like, while a few cells had polygonal or oval nucleus. The result suggested that most of SHEDs were stem cells mixed with other cell types (Figure 1A). To further validate whether these SHEDs were stem cell-like, two cell markers, cytokeratin and vimentin, were examined by immunochemistry. The cell marker of differentiated epithelial cells, cytokeratin was negative (Figure 1A). On the other hand, the immune staining of vimentin was strongly expressed in the nucleus of SHEDs (Figure 1A). During third passage culture, SHEDs entered the exponential growth phase on the second day and stationary phase on the 6th to 7th day based on the growth curve (Figure 1B), indicating that extracted SHEDs had a high proliferation potential. Cell surface markers CD34, CD44 and CD105 were checked by flow cytometry. Ninety-nine percent of the cells were CD44-positive; 90.13% were CD105-positive; all of the cells lacked hematopoietic markers CD34 (Figure 1C), further confirming the high proliferation potential. The above results demonstrated that the SHEDs were mostly MSCs.

### Extracted SHEDs had high differentiation potential

**Figure 2:**
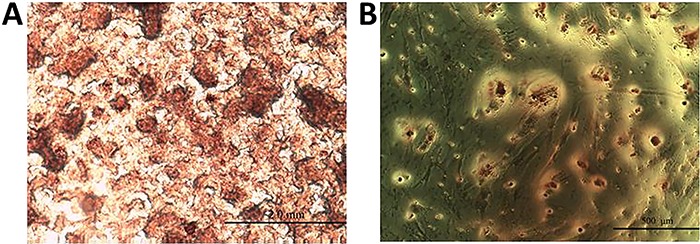
Stem cells from human exfoliated deciduous teeth presented a high differentiation capacity during adipogenesis and mineralization. *A*, cells after 30 days treatment with mineralization induction medium. Cells were stained with Alizarin red,×10. *B*, cells after 21 days treatment with adipogenesis induction medium. Cells were stained with oil red-O,×100.

Subsequently, we assessed SHEDs *in vitro* osteogenic and adipogenic differentiation. SHEDs were harvested and treated with mineralization-inducing medium. During the first six days, the cells grew rapidly and gradually overlapped each other. On the 30th day, clear distinct nodules were detected in the center of the cell and Alizarin red staining was positive under a microscope (Figure 2A). Consistent results were obtained in the cell adipogenesis assay; the SHEDs formed a clear bright point with positive oil red-O staining on the 21st day after adipogenesis-inducing medium culture (Figure 2B). The results demonstrated that SHEDs were not only proliferative but also had a high differentiation potential.

### Effect of different concentrations of mPRP on SHEDs proliferation

**Figure 3:**
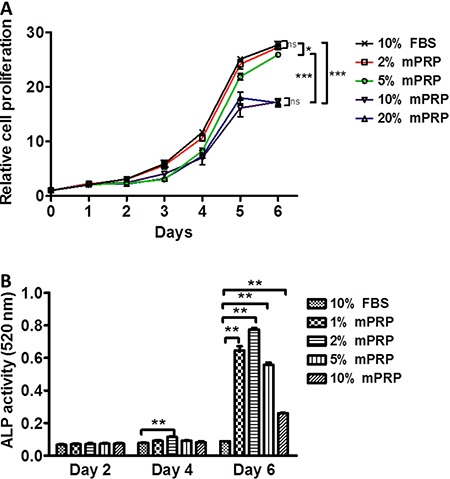
Effect of different concentrations of modified platelet-rich plasma (mPRP) on cell proliferation (*A*) and ALP activity of stem cells from human exfoliated deciduous teeth (*B*). Data are reported as means±SD from three independent experiments. *P<0.05, **P<0.01, ***P<0.001 *vs* control group (10% FBS) (Student's t-test).

Improved rich PRP was a pale yellow liquid. Platelet concentration was 10^12^/L, about five times higher than normal. mPRP was enriched with various growth factors. The concentration of PDGF-AA was 19.159 µg/L and the concentration of TGF-β1 was 57.163 µg/L (data not shown). Figure 3A shows the effect of different concentrations of mPRP on SHEDs proliferation. At the beginning of cell culture, 2, 5, 10, and 20% of mPRP had no significant improvement on SHEDs proliferation compared with the control group. From day 3 to day 5, SHEDs in all groups entered the exponential growth phase. Two percent mPRP showed a similar promotion effect as 10% FBS. The proliferation for 5% mPRP was a little lower than that of 2% mPRP and 10% FBS. However, 10 and 20% mPRP exhibited a much weaker proliferation (P<0.05) when compared with the control, 2 and 5% mPRP. When the cells reached a stationary phase on day 6, 2 and 5% mPRP-treated groups had a similar number of SHEDs similar to that of the 10% FBS group, but 10 and 20% mPRR had less cells compared with the above groups. These results indicated that different concentrations of mPRP improved SHEDs proliferation dose-dependently. Two percent mPRP seems to be the ideal culture concentration for SHEDs growth.

### Effect of different concentrations of mPRP on SHEDs mineralization

ALP is an important component during SHEDs mineralization. Thus, we determined the levels of SHEDs mineralization by checking ALP activity. Although different concentrations of mPRP did not show a superior promoting effect on SHEDs proliferation than 10% FBS, they caused a striking upregulation of ALP activity on the 6th day of SHEDs culture (P<0.01; Figure 3B). A concentration increase from 1 to 2% mPRP activated the ALP activity. This increase reached a peak at 2% mPRP, as a concentration increase from 2 to 10% decreased the ALP activity. Compared with day 2 and day 4, day 6 SHEDs showed the highest ALP activity, which suggests that when SHEDs reach confluence at stationary phase, the cells enter the mineralization differentiation period.

### Effect of different concentrations of mPRP's on SHEDs differentiation factors

**Figure 4:**
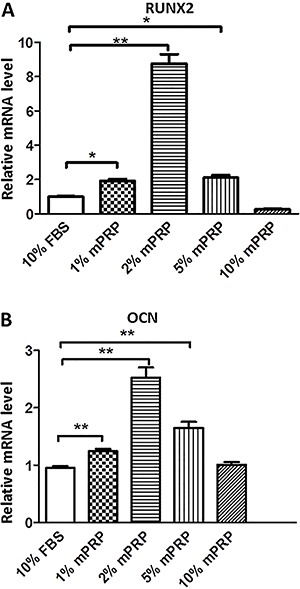
Effect of different concentrations of modified platelet-rich plasma (mPRP) on mRNA expression of RUNX2 (*A*) and OCN (*B*) in stem cells from human exfoliated deciduous teeth (SHEDs) cells, by quantitative real-time PCR. *P<0.05, **P<0.01 *vs* control group (Student's t-test).

RUNX2 and OCN are two key factors that lead to MSC osteoblast differentiation. Therefore, we focused on these genes during SHEDs osteoblastic differentiation induced by mPRP treatment. Real-time PCR results showed that on day 7 after mineralization media induction, 1, 2, and 5% mPRP significantly upregulated the mRNA expression of RUNX2 compared with the control group. A concentration of 10% mPRP conversely repressed the RUNX2 levels (Figure 4A). A similar result was obtained for OCN mRNA levels, which were significantly induced in the 1, 2 and 5% mPRP groups, but not in the 10% mPRP group compared with the control group (Figure 4B).

## Discussion

Even though mPRP has been widely used in clinical applications for decades because of its enriched autologous growth factors and secretory proteins, few studies have focused on its influence on SHEDs proliferation and differentiation. Furthermore, little has been done to find a better alternative to 10% FBS when culturing SHEDs.

In the present study, we successfully isolated and purified mPRP with high quality. As several reports have shown, PRP effects vary among individuals due to age-related systemic feedback mechanisms and different serum supplements (19,20). Therefore, we mixed four batches of mPRPs to reduce individual variation. Platelets from AB blood type were used to minimize antibody components, which could repress agglutination caused by immune rejection. To reduce the contamination of other cell components during mPRP separation, multifunctional cell separator was applied to the collected mPRP. Moreover, we also utilized the improved mPRP activation technology by repeated liquid nitrogen freezing and thawing. The various growth factors present in activated mPRP play important roles in cell proliferation, chemotaxis, and angiogenesis.

The extracted SHEDs were characterized with stem cell-like morphology. The cell growth curve proved that SHEDs were highly proliferative. Immunohistochemical results showed that SHEDs were MSC-like, cytokeratin-negative and vimentin-positive. Consistently, flow cytometry assay also showed that SHEDs were positively stained with MSC cell surface markers, CD44 and CD105. *In vitro* differentiation assay showed that SHEDs have a high potential to differentiate into osteoblasts and adipocytes.

To evaluate the possibility of replacing FBS by mPRP as a culture supplement during SHEDs growth and differentiation, we demonstrated that PRP promotion effect on SHEDs proliferation and differentiation was dose-dependent. The experiments proved that 2% mPRP had the optimal effect on the stem cells' proliferation. We also showed that an excessively high concentration of mPRP would impair the promotion effect. In a former study, we have explained that such a phenomenon is a result of prostaglandin E2 release by mPRP. Low concentrations of prostaglandin E2 can promote SHEDs proliferation while high levels will inhibit it (13). Another alternative explanation is related to the antiplatelet growth factor component in the plasma (10,21). Flow cytometry assay showed that MSCs expressed high levels of these growth factor receptors, such as platelet-derived growth factor A and B and TGF-β1 receptors (9). The above growth factors can activate multiple SHEDs signaling pathways, such as PI3K and NFκB, further promoting cell regeneration and inhibiting apoptosis (1,17,22,23). A detailed screening on PRP components demonstrates that platelet-derived growth factor and insulin-like growth factor-1 promote cell proliferation; acidic fibroblast growth factor, insulin-like growth factor 1 and insulin-like growth factor 2 promote extracellular matrix synthesis; TGF-β, platelet-derived growth factor, acidic fibroblast growth factor and basic fibroblast growth factor are involved in DPSCs odontoblast differentiation (9,14,24).

Upregulation of OPG and ALP occurs during MSCs osteogenic differentiation (18). If mPRP differentiation medium could also promote osteogenic differentiation of SHEDs, its use in tissue engineering has full potential, presenting the desired effect and avoiding risks such as infection and immune rejection. ALP activity is an important indicator for osteoblast cell differentiation and maturation, reflecting the level of mineralization ability and osteogenic transformation (25). Our result suggests that different concentrations of mPRP can enhance ALP activity of SHEDs, with an optimized concentration of 2%. RUNX2, which belongs to RUNX transcriptional factor family, is a major gene that regulates a large number of critical genes during osteoblastic differentiation and skeletal morphogenesis. OCN, one of the targets of RUNX family, is an osteoblast-specific protein which is essential for bone cell maturation. Shen et al. (26) has found that *in vitro* culture of SHEDs can express osteoblast markers, such as RUNX2, OCN, and bone sialoprotein. As in former reports, we have confirmed the upregulation of RUNX2 and OCN during SHEDs osteogenesis. These results indicate that during SHEDs differentiation, mPRP had an advantage over 10% FBS through enhancement of ALP activity and upregulation of mineralization factors, RUNX2 and OCN.

We showed that mPRP contains a high concentration of PDGF-AA and TGF-β1. mPRP dose-dependently improved SHEDs proliferation similar to 10% FBS and it has a superior function in promoting SHEDs osteogenesis. Notably, as SHEDs would not cause antigen-induced immune rejection, they could be applied in allograft and in dental tissue engineering. mPRP could serve as an alternative to replace FBS in *in vitro* culture and differentiation of SHEDs, which could hopefully resolve the current challenge in SHEDs amplification and improve the clinical safety during dental regenerative therapy.
